# Supplementary material for: Lactobacillus delbrueckii subsp. bulgaricus 2038 and Streptococcus thermophilus 1131 Induce the Expression of the REG3 Family in the Small Intestine of Mice via the Stimulation of Dendritic Cells and Type 3 Innate Lymphoid Cells
Source: Nutrients. 2019 Dec 7;11(12):2998. doi: 10.3390/nu11122998 (PMC6950248; doi:10.3390/nu11122998)
Supplement: Supplementary file 1 [file nutrients-11-02998-s001.pdf]

Supplementary materials and methods

1. Mice

Eight-week-old SPF male BALB/c mice were purchased from Japan SLC and used for the preparation of peritoneal macrophages. All mice were housed with group breeding and fed CRF-1 (Oriental Yeast, Tokyo, Japan), a purified diet. For euthanasia, cervical dislocation was conducted.

All study protocols were approved by the Animal Experimental Committee of Meiji Co., Ltd. (approval no. 2016_3871_0240, approval date December 20, 2016). The experiments were performed according to the guidelines of the committee.

2. In vitro experiment of IL-23 production using peritoneal macrophages

Peritoneal macrophages were collected from the peritoneal cavity of BALB/c mice as described previously [1]. Briefly, 2 mL sterile 4% Brewer thioglycollate medium (Becton Dickinson, Cockeysville, MD, USA) was intraperitoneally injected to mice. After four days, peritoneal macrophages were collected by washing the peritoneal cavity with 5 mL ice-cold PBS (pH 7.4).

Peritoneal macrophages were cultured in RPMI 1640 containing 10% FBS, 100 U/mL penicillin, and 100 μg/mL streptomycin at a concentration of 5.0×10^4 cells/mL in a 96-well culture plate (0.1 mL/well) at 37 °C in 5% CO_2. One microliter of PBS or heat-killed *L. bulgaricus* 2038 or *S. thermophilus* 1131 was added in medium and incubated for 24 h. The culture supernatants were collected for the determination of IL-23 levels.

1. Zhang, X.; Goncalves, R.; Mosser, D.M. The isolation and characterization of murine macrophages**.** *Curr. Protoc. Immunol.* **2008**, Chapter 14**,** Unit 14.11, doi:10.1002/0471142735.im1401s83.

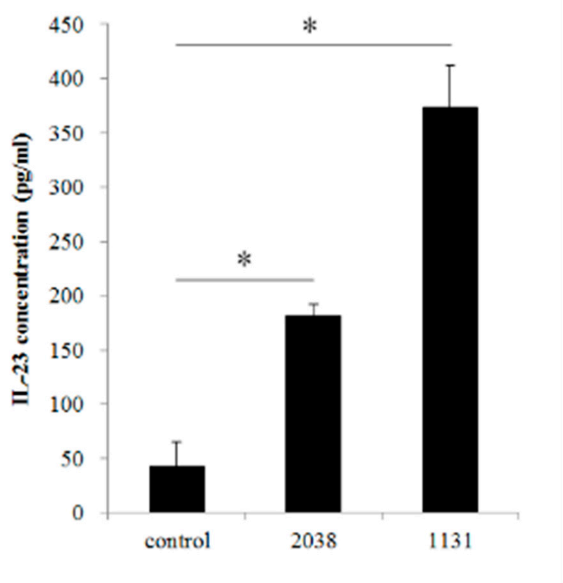

Figure S1. *L. bulgaricus* 2038 and *S. thermophilus* 1131 induced IL-23 production from peritoneal macrophages. Peritoneal macrophages were cultivated in the presence of heat-killed *L. bulgaricus* 2038 and *S. thermophilus* 1131 for 24 h, and supernatants were determined for IL-23 by ELISA (n = 3 per group). Data are mean ± SE. *$p < 0.05$.
